# *De novo* genome assembly and functional annotation for *Fusarium langsethiae*

**DOI:** 10.1186/s12864-022-08368-0

**Published:** 2022-02-22

**Authors:** Ya Zuo, Carol Verheecke-Vaessen, Corentin Molitor, Angel Medina, Naresh Magan, Fady Mohareb

**Affiliations:** 1 The Bioinformatics Group, Cranfield Soil and Agrifood Institute, School of Water, Energy and Environment, Cranfield University, College Road, MK43 0AL Bedford, UK; 2 Applied Mycology Group, Environment and AgriFood Theme, Cranfield University, College Road, MK43 0AL Bedford, UK

**Keywords:** Mycotoxins, T-2, HT-2, Oats, Trichothecenes, Long reads

## Abstract

**Background:**

*Fusarium langsethiae* is a T-2 and HT-2 mycotoxins producing species firstly characterised in 2004. It is commonly isolated from oats in Northern Europe. T-2 and HT-2 mycotoxins exhibit immunological and haemotological effects in animal health mainly through inhibition of protein, RNA and DNA synthesis. The development of a high-quality and comprehensively annotated assembly for this species is therefore essential in providing the molecular understanding and the mechanism of T-2 and HT-2 biosynthesis in *F. langsethiae* to help develop effective control strategies.

**Results:**

The *F. langsethiae* assembly was produced using PacBio long reads, which were then assembled independently using Canu, SMARTdenovo and Flye. A total of 19,336 coding genes were identified using RNA-Seq informed *ab-initio* gene prediction. Finally, predicting genes were annotated using the basic local alignment search tool (BLAST) against the NCBI non-redundant (NR) genome database and protein hits were annotated using InterProScan. Genes with blast hits were functionally annotated with Gene Ontology.

**Conclusions:**

We developed a high-quality genome assembly of a total length of 59 Mb and N50 of 3.51 Mb. Raw sequence reads and assembled genome is publicly available and can be downloaded from: GenBank under the accession JAFFKB000000000.

All commands used to generate this assembly are accessible via GitHub: https://github.com/FadyMohareb/fusarium_langsethiae.

**Supplementary Information:**

The online version contains supplementary material available at 10.1186/s12864-022-08368-0.

## Background

*Fusarium langsethiae* is a fungus belonging to the family *Nectriaceae*. It commonly infects ripening oats without showing any visible symptoms especially in Northern Europe, and contaminates the grains with the type A trichothecenes, T-2 and HT-2 [1, 2]. These mycotoxins mainly inhibit proteins, RNA and DNA synthesis leading to immunological and haematological effects [3].

Originally, this species was considered to be a “powdery *F. poae*”. However, subsequently it was shown to be a separate species and classified as *F. langsethiae* in 2004 by Torp and Nirenberg [2]. More than 300 *Fusarium* species exist [4] with different ecophysiological responses to parameters such as temperature and water availability. *F. langsethiae* is a relatively slow colonizer of temperate cereals although the ability to produce mycotoxins may provide some competitiveness in the phyllosphere microbiome [5, 6].

Some other more economically important *Fusarium* species, e.g. *F. graminearum*, have received more attention as they produce visible symptoms in wheat (Fusarium head blight) and have thus been widely sequenced and analyzed previously [7].

The species in the *Fusarium* family are known to produce a wide range of secondary metabolites including type A and type B trichothecenes and zearalenone. Most significant strides have been made in relation to an understanding of the role of gene clusters involved in type B trichothecenes and fumonisins, which are produced predominantly by *F. graminearum*/*F. culmorum* and *F. verticillioides*/*F. proliferatum*, respectively [8]. In addition, the former species also produces other mycotoxins, such as Zearalenone, Fusaric acids and Moniliformin especially in temperate cereals [9]. However, legislation on contamination of temperate cereals is predominantly focused on type B trichothecenes such as deoxynivalenol and zearalenone. Based on substitutions at C-8 and other positions around the core structure, trichothecenes have been classified into four groups (types A, B, C and D) [8], *Fusarium* species only produce type A and type B. Type A, which have an ester or hydroxyl or no oxygen substitution in the position of C-8, are usually produced by *F*. *armeniacum*, *F*. *langsethiae*, *F. poae*, *F*. *sambucinum*, *F*. *sporotrichioides*, and *F*. *venenatum*. Type B, which have a carbonyl group at C-8, are mainly produced by *F. graminearum* and *F. pseudograminearum* and *F. culmorum*. Type A compounds are generally more toxic than type B trichothecenes while the latter are usually produced in higher concentration.

It should be noted however that, many of these studies have focused on the toxin biosynthesis process [10, 11]. Some of these studies use sequencing methods to analyse protein structure or toxin-related biosynthetic gene clusters. However, most of the *Fusarium* sequences comes from short reads platforms, which may have thus excluded some important functional gene clusters and their proteins.

The aim of this work was to produce a high quality, deep coverage genome assembly for *F. langsethiae* through a long-reads assembly strategy using the PacBio platform. This allowed us to generate a more complete, continous genome assembly compared to the previous publicly available *F. langsethiae* assembly. Further analysis including identification of the clusters related to biosynthesis of T-2 and HT-2 mycotoxins was also examined. The relatively small length of fungal genomes makes them ideal candidates for long-reads assembly, since it is possible to achieve deep read coverage, allowing a higher degree of continuity with no noticeable implication on the sequencing costs.

## Results

Since this species had not been previously assembled using long-reads, different assemblers were compared to achieve a higher quality overall assembly. After comparing each assembly’s statistical information and BUSCO results, the best assembly was polished as the final assembly. Assembly quality metrics for all five chosen are shown in Table 1, which outlines some basic statistical data from the different assemblies. The total assembly lengths obtained from Canu [12] and Flye [13] were relatively closer to each other compared to SMARTdenovo [14], which had the longest genome length and the largest number of contigs, indicating fragmentation. Most of the GC content results in this study were around 48.4%; the SMARTdenovo assembly was slightly lower. The highest N50 value was achieved by Flye, which was nearly five times longer than the best Canu assembly and 22 times longer than the SMARTdenovo assembly.

**Table 1:** Basic statistic information of draft assembly from Canu, SMARTdenovo and Flye

Assembly method		Canu		SMART denovo	Flye
Corrected error rate	0.045	0.065	0.085	-	-
Contigs	301	301	280	277,164	177
Contigs (>=50 kb)	174	174	168	170	60
Total contigs length	62,453k	62,451k	62,870k	3,850,300k	59,663k
Total contigs length (>=50 kb)	59,418k	59,416k	59,802k	9,340k	59,004k
Longest contig	2,495k	2,495k	2,723k	77k	11,601k
GC content (%)	48.46	48.48	48.44	48.21	48.43
N50	512k	521k	614k	16k	3,513k
#N’s per 100 kbp	0.0	0.0	0.0	0.0	0.0

The draft assembly from Flye achieved 98.3% completeness, as assessed by BUSCO, and had fewer fragmented and missing hits compared with Canu (See Table 2). On the other hand, the SMARTdenovo assembly only had 11 complete hits, probably due to the fragmented state of this assembly. Considering the contigs length, N50 and BUSCO [15] results, Flye output was considered the best assembly which was then carried forward for error correction.

**Table 2:** BUSCOs statistic result from Canu, SMARTdenovo and Flye

Assembler	Canu			SMART-denovo	Flye
Corrected error rate	0.045	0.065	0.085	-	-
complete hits (%)	90.8	90.9	91.7	0.3	98.3
Complete hits no.	3,384	3,387	3,417	11	3,659
Complete single copy	3,361	3,364	3,393	11	3,642
Complete duplicated	23	23	24	0	17
Fragmented hits	151	150	148	34	48
Missing hits	190	188	160	3,680	18

To improve the assembly quality further, error correction was performed with Pilon based on the previously mentioned publicly available Illumina short reads. Comparing the statistical sequence information and BUSCO results between draft assembly and polished assembly, the length of contigs did not change after hybrid polishing, but the BUSCO results improved from 98.3 to 98.8% (See Table 3). Moreover, 28 of the fragmented genes became either complete [20] or missing [8], which support the fact that Pilon actually removed mis-assemblies from the raw draft. Compared with the previous assembly of another *F. langsethiae* strain (Fl201059), the draft assembly of this study has better contiguity and slightly higher BUSCO results. To finish the polishing, 23 contigs were detected as mitochondrial hits and were removed from the assembly.

**Table 3:** Basic sequence statistic information and BUSCO result comparation between before polishing and after polishing

Draft assembly	Previous assembly (Lysoe, 2016)	Flye Before polishing	Flye After polishing
Contigs	1,586	177	154
Total length	37,543,021	59,662,685	59,637,819
Largest contig	829,859	11,601,651	11,601,651
N50	86,515	3,513,144	3,513,144
Complete BUSCO hits (%)	98.2	98.3	98.8
Complete	3659	3659	3679
Complete single copy	3,647	3,642	3,661
Complete duplicated	12	17	18
Fragmented	24	48	20
Missing	42	18	26

RepeatMasker was deployed using the default parameters, which was followed by RepeatModeller in order to perform de-novo identification and classification of transposable elements (TE). Retroelements represented 4.70% of the total assembly sequence, while DNA transposons formed 21.02%. This figure is comparable to what has been previously reported for *F. poae* [16, 17]. The detailed classification of repeats and TE is provided in Supplementary File 1.

A series of gene prediction approaches were followed using different settings or alignment models from related species as shown in Table 4. A hints file was created for Augustus using the cDNA sequence of sample Fl201059 downloaded from EMBL-EBI [18]. Firstly, the cDNA contigs were aligned to the assembled genome in order to confirm its suitability. A total of 15,280 alignments were obtained, representing 96% mapping results which confirms its suitability to guide the gene prediction process. GeneID in ab-initio mode predicted 21,848 genes, while Augustus predicted a total 16,900 genes. Then, after training, Augustus predicted 19,336 genes.

**Table 4:** Number of predicted genes from different tools, settings, and reference species

Tools	GeneID		Augustus		
Setting	*Ab Inito*		With hints	Training	
Reference species	*F.* *oxysporum*	*F.* *graminearum*	*F. langsethiae* (hints),*F. graminearum* (model)	*F. langsethiae*	
Predicted genes	21,848	18,403	17,638	19,336	

Following the gene prediction step, a BLAST search was performed of predicted coding genes against the NR database, and the hits were further annotated with GO terms and protein signatures. A total of 19,139 out of 19,336 predicted genes (98.98%) had more than five hits against the NR database. This meant that more than 99% of genes predicted by Augustus were reliable for protein analysis to some extent. Among the hits, most had a similarity percentage higher than 80%; some of them reached 100% similarity with the sequence in the NR database.

More than 50% of the genes in the assembly had top hits compared with *F. langsethiae* itself. Almost all of these were within the *Fusarium* genus. Table 5 shows the top 10 BLAST hits distribution, all of them coming from the *Fusarium* genus. The assembly showed a linkage between *F. langsethiae* and *F. poae*, *F. oxysporum, F. graminearum*, *F. sporotrichioides* therefore indicating a similarity in the metabolic pathways and/or mycotoxin production. As the *F. poae* had the highest number of hits amongst all the *Fusarium* species, it suggests a close relevance between *F. langsethiae* and *F. poae*. Indeed, it should be considered that in papers published before 2004, *F. langsethiae* was considered as “powdery *F. poae”.* It could be inferred that, *F. poae* should thus have the closest linkage to *F. langsethiae*, amongst all the *Fusarium* species.

**Table 5:** Top 20 blast top hits distribution among predicted genes

Species	Top hits
*Fusarium poae*	*50,396*
*Fusarium oxysporum*	*33,516*
*Fusarium oxysporum f. sp. cepae*	*25,207*
*Fusarium graminearum*	*24,162*
*Fusarium langsethiae*	*15,412*
*Fusarium oxysporum f. sp. cubense*	*14,995*
*Fusarium fujikuroi*	*11,795*
*Fusarium sporotrichioides*	*11,391*
*Fusarium oxysporum f. sp. narcissi*	*11,259*
*Fusarium venenatum*	*11,110*

Proteins related to trichothecene and HC-Toxin are shown in Tables 6 and 7 respectively. These tables list the contigs in which each protein was located, as well as the similarity to genes in other species as found via BLAST. The ontology term and ontology ID give a basic description of the protein and its functions.

### *TRI* genes

**Fig. 1:**
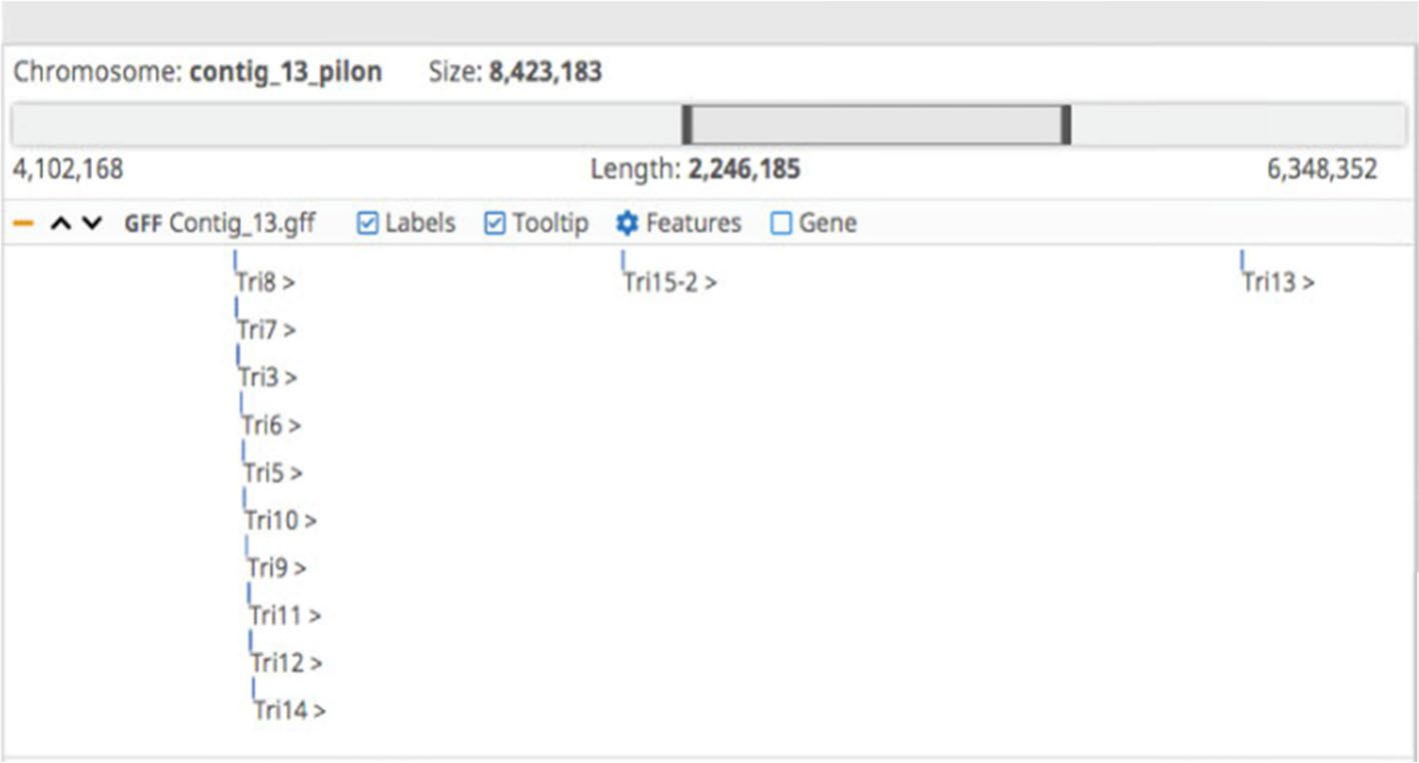
TRI-genes cluster identified on Contig 13 of the assembly

Table 6 lists gene hits related to the *TRI* genes cluster. In the BLAST stage, 33 sequences were found. Most of them were gathered at the thirteenth contig of our assembly (as shown in Fig. 1), while some were gathered at the fifty-third and fifty-seventh contigs amongst others.

**Table 6:** Gene position, for the *TRI* gene cluster

Contig	Position	Description of protein activity	Gene
contig_13	4474108-4475891	3-acetyltrichothecene 15-o-acetyltransferase	*TRI3*
	4482462-4483646	aChain A, Trichodiene Synthase	*TRI5*
	4496454-4498287	cytochrome P450 monooxygenase	*TRI1*
	6073702-6075258	dtdp-glucose 4,6-dehydratase	*TRI13*
	4476651-447839	P450 monooxygenase	*TRI4*
	6196131-619846	protein phosphatase 2 (formerly 2 A)	*TRI4*
	4479511-4480165	regulatory protein	*TRI6*
	4484262-4485602	regulatory protein	*TRI10*
	4498937-4500113	TRI14	*TRI14*
	5087905-5088817	TRI5-2 protein	*TRI5-2*
	4488333-4488662	TRI9	*TRI9*
	4471969-4473348	trichothecene-4-O-acetyltransferase	*TRI7*
	4491419-4493141	trichothecene c-15 hydroxylase	*TRI11*
	4469927-4471271	trichothecene c-3 deacetylase	*TRI8*
	4493646-4495682	trichothecene efflux pump	*TRI12*
contig_53	7949550-7951359	c6 transcription factor	A0A2L2*TRI1*
	413927-415700	cytochrome p450 monooxygenas	*TRI1*
	8051646-8052534	related to TRI15-putative transcription factor	2146
	7113258-7114437	related to TRI7-trichothecene biosynthesis gene cluster	FIE12Z_415
	428032-429514	trichothecene C-8 acyl transferase	*Tri16*
contig_3	2237847-223899	AChain A, Trichodiene Synthase	*Tri5*
	582527-584300	cytochrome p450 monooxygenase	*Tri1*
	2006343-2010297	related to TRI13-cytochrome P450	Focb16_v015293
contig_57	4993092-4994992	CRAL-TRIO domain-containing protein C3H8.02	FVEG_12149
	5234170-5235550	richothecene 3-o-acetyltransferase	*Tri101*
contig_6	30398-32815	cytochrome p450	*Tri13*
contig_3	582527-584300	cytochrome p450 monooxygenase	*Tri1*
	2006343-2010297	related to TRI13-cytochrome P450	abd-A
contig_12	674307-676300	cytochrome P450 monooxygenase	*Tri1*
contig_74	539307-539832	cytochrome P450 monooxygenase	*Tri1*
	563537-564065	related to TRI13-cytochrome P450	abd-A
contig_37	225032-226812	related to TRI13-cytochrome P450	BFJ72_g11013
contig_92	972537-973648	related to TRI15-putative transcription factor	FPOA_05731

### HC-toxin related genes

**Fig. 2:**
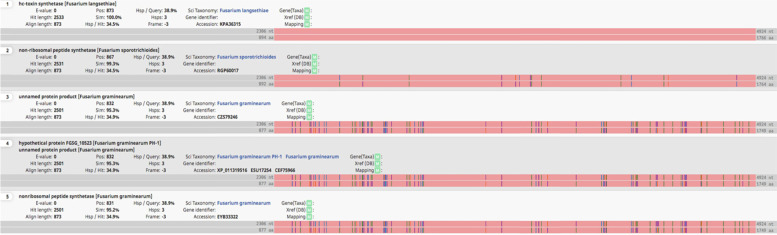
Blast hits of the hc-toxin gene highlighting 14 a.a.changes compared to the closest blast hit of *F. sporotrichioides*

Five proteins were found to be related to HC-toxin; all of them are listed in Table 7; Fig. 2. Unlike the previous assembly, we identified two copies of HC-toxin synthetase located on the same contig and only 25 bases apart. Three proteins acted as an HC-toxin efflux carrier TOXA, as shown in Table 7.

**Table 7:** Gene position, similarity gene and description of genes related with the keyword “hc-toxin” in assembly annotation

Contig	Position	Description	Gene
contig_11	265115-271850	hc-toxin synthetase (non-ribosomal peptide synthetase)	KPA36315
	271875-272769	hc-toxin synthetase (non-ribosomal peptide synthetase)	KPA36315
contig_13	989078-990512	hc-toxin efflux carrier	Efflux pump roqT
contig_34	770120-771587	hc-toxin efflux carrier	Efflux pump roqT
contig_93	1020288-1023864	hc-toxin efflux carrier	Efflux pump roqT

### Related secondary metabolite genes

In addition, some contigs were linked to specific genes which are involved in global secondary metabolite biosynthesis. These include aldehyde reductase member 3 (Contig 13) and an efflux pump (Contig 2, 13, 57), ketose reductase (Contig 6, 13).

### Polyketide synthases (PKS) and non-ribosomal peptides (NRPS)

Polyketides (PKS) are a group of secondary metabolites with diverse functions, which in fungi can play a role in antifungal activity, for example to compete with other fungal species within the same ecosystem [19]. We have identified a total of 114 NRPS-related genes (See Supplementary Table 1) and a further 24 hits for PKS genes (See Table 8) compared to 12 PKS genes previously identified in *F. langsethiae* Fl201059 [18].

**Table 8:** Gene position, similarity gene and description of genes related with the keyword “PKS” in assembly annotation

Contig	Position	Description	Gene
contig_57	5846016-5857862	fusarin C cluster-polyketide synthase/NRPS	PKS10
contig_12	845216-851362	polyketide synthase	PKS8
contig_17	191466-199960	polyketide synthase	PKS8
contig_17	402469-402990	polyketide synthase	PKS11
contig_3	414213-416367	polyketide synthase	PKS8
contig_3	418532-419857	polyketide synthase	PKS8
contig_3	421866-423324	polyketide synthase	PKS8
contig_44	2246352-2252766	polyketide synthase	PKS11
contig_5	831960-835644	polyketide synthase	PKS8
contig_5	835726-839989	polyketide synthase	PKS8
contig_5	841956-842226	polyketide synthase	PKS8
contig_53	27347-29588	polyketide synthase	PKS8
contig_53	5950461-5958042	polyketide synthase	PKS14
contig_53	6570175-6572762	polyketide synthase	PKS6
contig_53	6572851-6578043	polyketide synthase	PKS6
contig_53	7712341-7718826	polyketide synthase	PKS12
contig_57	670938-671526	polyketide synthase	PKS8
contig_57	6166489-6175054	polyketide synthase	PKS6
contig_6	2457484-2464977	polyketide synthase	PKS7
contig_7	14743-15954	polyketide synthase	PKS8
contig_89	532944-541439	polyketide synthase	PKS8
contig_2	169007-171644	putative polyketide synthase	PKS11
contig_44	2231055-2232789	putative polyketide synthase	PKS5
contig_57	2601524-2608661	putative polyketide synthase	PKS3

### Mating-type genes (MAT) and cell wall degrading enzymes

*MAT* genes are controlling sexual reproduction and development in fungi [20]. Here, we identified 6 hits related to MAT including one transcription factor, MAT1-1-3 and MAT-1-1-1 (See Table 9). Additionally, three cuticle-degrading protease on three different contigs were also found (See Table 10).

**Table 9:** Gene position, similarity gene and description of genes related with the keyword “MAT” in assembly annotation

Contig	Position	Description	Gene
contig_57	1296199-1298787	hypothetical protein FLAG1_01022	MAT1
contig_6	2816292-2817495	mating type protein 1-1-1	MAT-1-1-1
contig_6	2812800-2813551	mating type protein 1-1-3	MAT-1-1-3
contig_92	578071-578908	transcription factor	MAT10
contig_13	3352399-3353403	ubiquinol-cytochrome-c reductase cytochrome c1	MATA1
contig_13	3380740-3382459	ubiquinol-cytochrome-c reductase cytochrome c1	MATA1

**Table 10:** Gene position, similarity gene and description of genes related with the keyword “Cuticle-degrading” in assembly annotation

Contig	Position	Description	Gene
contig_57	3160828-3161413	cuticle-degrading protease	FLAG1_04065
contig_13	4110892-4112114	Cuticle-degrading protease	FLAG1_07867
contig_33	96122-97344	Cuticle-degrading protease	FLAG1_07867

## Discussion and conclusions

With third-generation sequencing, the long reads and high depth generated using the PacBio^®^ SMRT sequencing led to a very high-quality assembly. The quality was not determined according to the contig length or N50 alone; BUSCO was another parameter used to examine the quality of the assembly. Compared to the publicly available assembly of Fl201059 which did not show good quality in the scaffold statistical data (N50 = 86,515 vs. 3.51 Mb in our assembly), but it had a high BUSCO compared to the *Saccharomyceta* dataset, showing a level of completeness of 98.2% vs. 98.8% in our assembly which had both high-quality contigs and a high BUSCO rate. This means that more coding genes could be predicted by Augustus and GeneID. This provided hints to improve the accuracy of prediction. However, although the hints file could improve the accuracy, the core model in Augustus still came from *F. graminearum*, a related species. However, training Augustus produced a model based on the *F. langsethiae* sequence file. With the absence of an aligned BAM file or gene bank structure file from EnsembleFungi Fl201059, a FASTA file containing protein in the same sample was used to train the Augustus model. While the *ab initio* approach predicted genes with a model from a different species, it has less accuracy compared to the hints and training. Although the hints file improved the accuracy from *ab initio* to some extent, for some new species such as *F. langsethiae*, the accuracy from the hints file did not achieve the expectations of the analysis. Training Augustus with data that had 96% similarity with the assembly predicted more genes than *ab initio* and the hints file.

### Mycotoxin production pathways

T-2 and HT-2 toxins are type A trichothecenes which mainly accumulate in oats and can cause immunological or haemotological defects in animals and potentially humans [3].

Almost all proteins related to trichothecene biosynthesis are located on the 13th contig of the assembly, with an additional copy of *TRI5* on contig 3 (See Fig. 1).

Other proteins located in other contigs did not seem to have a core function in T-2 and HT-2 biosynthesis, and most of them encoded the transformation or production of TRI-proteins. HC-toxin synthetase was identified on contig 11 as two copies only 25 bps apart. Both copies were identical to the previous *F. langsethiae* assembly, but with 14 a.a. changes compared to the closest blast hit of the RGP60017 gene of *F. sporotrichioides* (See Fig. 2). It could be therefore inferred that contig 13 contains the main functional proteins regrouped in a cluster. However, one of the genes encoding a protein involved in HT-2 and T-2 mechanism called TRI1, were found in the contig 3, 12, 53 and 74.

In 2011, [21] described trichothecenes biosynthesis in the *Fusarium* species. Based on these findings, the biosynthesis process in *F. langsethiae* with different proteins could be inferred with the gene BLAST results and descriptions of proteins in *F. langsethiae* based on previous studies of other *Fusarium* species and the previous draft genome of *F. langsethiae* [18].

The first step consists in the cyclisation of farnesyl pyrophosphate, which is a primary metabolic intermediate [21] and is mediated by a trichodiene synthase encoded in the gene *TRI5* in the 13th contig as well as another hit identified on contig 3, suggesting its presence in two copies. *TRI5* is the core gene that mediates the biosynthesis of different trichothecenes (gi | 136,010 | sp | P13513.1 | TRI5_FUSSP), including T-2 toxin. The ontology term of this gene (GO:0045482) indicates its molecular function as a trichodiene synthase. Tri5 is involved in the catalysis of the following reaction: 2-trans, 6-trans-farnesyl diphosphate = diphosphate + trichodiene. The *TRI5* gene was first characterised in a *F. sporotrichioides* strain that produced T-2 toxin [22]. The trichodiene then goes through an oxygenation series catalysed by cytochrome P450 monooxygenase encoded by *TRI4* [21]. The *TRI4* gene (gi | 927,758,023 | gb | KPA41245.1) encodes a mono-oxygenase molecular function (GO:0004497) leading to the addition of four oxygens at C-2, C-3, C-11 and C-12, using C-13-epoxide to form the intermediate isotrichotriol [23]. Subsequently, isotrichodermol (C-3-OH) is converted to isotrichodermin (C-3-OR) via an acetyltransferase encoded by *TRI101* (gi | 927,756,670 | gb | KPA40029.1) in the fifty-seventh contig [24]. The toxicity of *Fusarium* trichothecenes should be effectively reduced with this step, which serves as a mechanism for the fungal self-protection other trichothecene-producing organisms [25]. TRI101 (gene located in contig 57) acts as part of the transferase activity (GO:0016747) that transfers an acyl group, other than aminoacyl, from one compound to another. Then, a second hydroxyl group is added to C-15, which is controlled by TRI11, encoded in the contig 13 [26].

TRI11 (gi | 927,758,018 | gb | KPA41240.1) works with a molecular function (GO:0016705)—an oxidation-reduction reaction in which hydrogen or electrons are transferred from each of two donors as well as an oxidation- reduction process (GO:0055114). After this TRI3 (gi | 927,758,024 | gb | KPA 41246.1) (GO:0043386) catalyses the acetylation of the 4-hydroxyl to form trichodermin and then, TRI13 protein (gi | 927,758,016 | gb | KPA41238.1) perform the same oxidation-reduction reaction as TRI11 in C-4 [27], followed by another acetylation process by TRI7 (gi | 927,758,025 | gb | KPA41247.1).

The next step of this process in *F. sporotrichioides* is the addition of a fourth hydroxyl group to C-8 by TRI1, followed by an addition of an isovaleryl moiety thanks to TRI16. Finally, the C-3 position loses the acetyl group via a TRI8-esterase step to produce T-2 toxin [28].

In this study, the *TRI1* gene was found in the 3rd and 53rd contigs, and there was about 15% sequence dissimilarity with the *TRI1* sequence (gi | 927,755,786 | gb | KPA39264.1) in the database. Since *TRI16* was not found, it might have been either mis-matched and mis-labeled by BLAST or missing from the assembly. The gene *TRI8* was also found (gi | 927,758,026 | gb | KPA41248.1) and was identified as encoding a triglyceride lipase activity (GO:0004806) and is involved in a reaction in which triacylglycerol + H_2_O = diacylglycerol + a carboxylate.

Most of the genes linked to T-2 mycotoxin production were found in this assembly, but the *TRI9* gene only had sequences for which no literature could be found to describe their function. However, *TRI9* has been found not only in *F. langsethiae* but also other *Fusarium* species, such as *F. sporotrichioides* and *F. graminearum*. It acts upon the integral component of the membrane (GO:0016021) and might be linked with the T-2 mycotoxin transport mechanism.

With regard to the genes identified in relation with the HC-toxin, not enough information was found to support the production pathways in *F. langsethiae*. However, some genes encoded global regulators such as the efflux pump and carrier of HC-toxin produced by *Cochliobolus* species might have an evolving relationship with *F. langsethiae*.

## Methods

### Sequencing Data

*F. langsethiae* Fe2391 strain was selected as this strain, originating from the UK, has been previously characterized as potent producer of T-2 and HT-2 toxin [6, 29].

To obtain long sequence fungal DNA, the protocol from Bacha [30] was used. Briefly, 3-day-old colonies of *F. langsethiae* Fe2391 grown on Potatoes Dextrose Agar (PDA) were harvested and frozen in liquid nitrogen. The mycelia were incubated for 10 min at 50 °C in a modified lysis buffer (1% of hexadecyl-trimethyl-ammonium bromide, 100mM pH8 EDTA, 1.4 M NaCl, 20mM pH8 Tris-HCl). The DNA was then extracted 3 times in phenol:chloroform prior to precipitation in isopropanol. The pellet was resuspended with 25U of RNAse prior to sending for sequencing. The DNA size (~20 kb) was validated by gel electrophoresis. The samples were sent to Novogene, China, which generated raw sequencing data using the PacBio^®^ Sequel platform.

### Genome Assembly

To achieve the best assembly quality possible, three separate assemblers were used to process the raw *F. langsethiae* data, namely Canu v1.8, SMARTdenovo, and Flye v2.4.2. Canu has three phases in its pipeline: correction, trimming and assembly. Since Canu is sensitive to the sequences’ genome size, it requires a parameter called ‘error rate’, which refers to the percentage of difference between the two reads in an alignment. The genome size parameter was set to 37,500,000 for this study, based on the length of the assembly publicly available for this species. According to Canus’ user guide, the parameters of error rate should be adjusted according to the coverage and data type of the raw data. Therefore, the error rates were set to 0.045, 0.055, 0.065, 0.075, 0.080, 0.085 and 0.095; this kept the correction, trimming and assembling stages identical. The second assembler used was SMARTdenovo (available at https://github.com/ruanjue/smartdenovo). This tool directly utilises reads from raw read alignments without correction or trimming phases, and it provides its own polishing methods to generate accurate consensus sequences. For the present study, the raw data were directly processed by the SMARTdenovo.pl script with all parameters set to default. The last, and the best performing assembler of the three was Flye, previously called A-Bruijn. It uses a repeat graph as its core data structure and utilises raw data in FASTA or FASTQ format from PacBio^®^. Flye outputs polished contigs with an error rate less than 30% by default. To run the assembly, the genome size was set to 37,500,000 and other parameters were set to default.

A total of five draft assemblies were generated using all three assemblers. QUAST, a quality assessment tool for genome assemblies [31], was used to examine the basic quality among the contig assemblies by comparing their total length, longest contig and N50 number. The best assembly output was identified according to completeness through orthologs comparison versus the *Saccharomyceta* OrthoDB [32] data set using the Benchmarking Universal Single-Copy Orthologs (BUSCO) tool [15]. BUSCO results were judged based on the number of complete BUSCO genes, and then the best assembly was used in the next stage.

To further improve the assembly, polishing was performed using Pilon [33]. Since short reads were absent for this sample, the WGS sequence reads of sample Fl201059 [18] were downloaded from the European Nucleotide Archive and aligned to our assembly using BWA-MEM [34]. This resulted in an alignment file in BAM format, which was then used by Pilon to perform error correction.

### Gene prediction and functional annotation

Two gene prediction methods, GeneID [35] and Augustus [36], were used with four separate procedures, GeneID *ab-inito*, Augustus *ab-inito*, Augustus with hints and Augustus with training. For *ab-initio*, Augustus predicted genes using *F. graminearum*, and GeneID predicted genes using *F. oxysporum*. Only Augustus had settings that could be used for hints prediction and training. The hints were created from the cDNA file from Fl201059. For training, the BRAKER pipeline [37] with the protein coding file from Fl201059 was used; this pipeline contains an automatic training and prediction pathway using GenomeThreader and Augustus.

A BLAST search [38] was performed to find regions of local similarity between sequences. Gene sequences that had been extracted from prediction tools were output in a GFF format and then made into a FASTA file. Using the blastx command, the FASTA file was compared with the NR nucleotide database. The number of threads was set to 50. The output format of this command was set as BLAST archive (ASN.1). Functional annotation was performed using OmicsBox (available at https://www.biobam.com/). The BLAST hits were imported into OmicsBox to perform Gene Ontology mapping and annotation. InterPro protein signatures and domain hits were obtained using InterProScan5. The output was then imported in OmicsBox and merged with the GO annotation and mapping results.

## Supplementary Information

**Additional file 1.**

**Additional file 2.**

## Data Availability

The genome assembly generated in this study as well as the raw sequence reads are available via the NCBI SRA repository under the Bioproject number: PRJNA701381 (Link: https://www.ncbi.nlm.nih.gov/nuccore/JAFFKB000000000).
