# Supplementary material for: De novo genome assembly and functional annotation for Fusarium langsethiae
Source: BMC Genomics. 2022 Feb 22;23:158. doi: 10.1186/s12864-022-08368-0 (PMC8864894; doi:10.1186/s12864-022-08368-0)
Supplement: Supplementary file 1 — Additional file 1. [file 12864_2022_8368_MOESM1_ESM.docx]

**Supplementary File 1: RepeatMasker output**

=================================================

file name: flye_assembly.fasta

sequences: 177

total length: 59663421 bp (59663421 bp excl N/X-runs)

GC level: 48.43 %

bases masked: 19249614 bp ( 32.26 %)

==================================================

number of length percentage

elements* occupied of sequence

--------------------------------------------------

Retroelements 2105 2807068 bp 4.70 %

SINEs: 0 0 bp 0.00 %

Penelope 0 0 bp 0.00 %

LINEs: 606 503352 bp 0.84 %

CRE/SLACS 0 0 bp 0.00 %

L2/CR1/Rex 0 0 bp 0.00 %

R1/LOA/Jockey 0 0 bp 0.00 %

R2/R4/NeSL 0 0 bp 0.00 %

RTE/Bov-B 0 0 bp 0.00 %

L1/CIN4 0 0 bp 0.00 %

LTR elements: 1499 2303716 bp 3.86 %

BEL/Pao 0 0 bp 0.00 %

Ty1/Copia 609 1539811 bp 2.58 %

Gypsy/DIRS1 171 607294 bp 1.02 %

Retroviral 0 0 bp 0.00 %

DNA transposons 9786 12543781 bp 21.02 %

hobo-Activator 4562 6296620 bp 10.55 %

Tc1-IS630-Pogo 4086 5027064 bp 8.43 %

En-Spm 0 0 bp 0.00 %

MuDR-IS905 0 0 bp 0.00 %

PiggyBac 104 44950 bp 0.08 %

Tourist/Harbinger 0 0 bp 0.00 %

Other (Mirage, 0 0 bp 0.00 %

P-element, Transib)

Rolling-circles 133 79452 bp 0.13 %

Unclassified: 9878 3495440 bp 5.86 %

Total interspersed repeats: 18846289 bp 31.59 %

Small RNA: 32 24498 bp 0.04 %

Satellites: 0 0 bp 0.00 %

Simple repeats: 6571 262884 bp 0.44 %

Low complexity: 777 36491 bp 0.06 %

==================================================

* most repeats fragmented by insertions or deletions

have been counted as one element

RepeatMasker version 4.1.2-p1 , default mode
